# Resonance Energy Transfer to Track the Motion of Lanthanide Ions—What Drives the Intermixing in Core-Shell Upconverting Nanoparticles?

**DOI:** 10.3390/bios11120515

**Published:** 2021-12-14

**Authors:** Philipp U. Bastian, Nathalie Robel, Peter Schmidt, Tim Schrumpf, Christina Günter, Vladimir Roddatis, Michael U. Kumke

**Affiliations:** 1 Institute of Chemistry (Physical Chemistry), University of Potsdam, 14469 Potsdam, Germany; philipp.bastian@uni-potsdam.de (P.U.B.); nathalie.robel@uni-potsdam.de (N.R.); peschmidt@uni-potsdam.de (P.S.); tschrumpf@uni-potsdam.de (T.S.); 2 Institute of Geosciences (Mineralogy), University of Potsdam, 14469 Potsdam, Germany; guenter@geo.uni-potsdam.de; 3 Helmholtz Centre Potsdam, GFZ German Research Centre for Geosciences, 14473 Potsdam, Germany; roddatis@gfz-potsdam.de

**Keywords:** upconversion nanoparticles, lanthanoid migration, lanthanides, core-shell, energy transfer

## Abstract

The imagination of clearly separated core-shell structures is already outdated by the fact, that the nanoparticle core-shell structures remain in terms of efficiency behind their respective bulk material due to intermixing between core and shell dopant ions. In order to optimize the photoluminescence of core-shell UCNP the intermixing should be as small as possible and therefore, key parameters of this process need to be identified. In the present work the Ln(III) ion migration in the host lattices NaYF_4_ and NaGdF_4_ was monitored. These investigations have been performed by laser spectroscopy with help of lanthanide resonance energy transfer (LRET) between Eu(III) as donor and Pr(III) or Nd(III) as acceptor. The LRET is evaluated based on the Förster theory. The findings corroborate the literature and point out the migration of ions in the host lattices. Based on the introduced LRET model, the acceptor concentration in the surrounding of one donor depends clearly on the design of the applied core-shell-shell nanoparticles. In general, thinner intermediate insulating shells lead to higher acceptor concentration, stronger quenching of the Eu(III) donor and subsequently stronger sensitization of the Pr(III) or the Nd(III) acceptors. The choice of the host lattice as well as of the synthesis temperature are parameters to be considered for the intermixing process.

## 1. Introduction

Upconversion nanoparticles (UCNP) are potential optical probes for many applications in the environmental and life science context. In order to bring UCNPs into a broad practical application, further improvements in the synthesis design, host lattices, stability in water, and surface functionalization are needed to meet the specific challenges of real-world applications. UCNPs are competing with established optical probes such as organic dyes or quantum dots. Here, a major issue is the low brightness of UCNPs which limits their use in practical applications [1,2,3,4,5,6], e.g., for imaging, diagnostics and therapy (theranostics) [5,6,7,8,9]. UCNPs with at least one (protective) shell around the nanoparticle core, which contains the sensitizer and activator ion, is a very frequently used strategy to improve the UCNP emission efficiency. Here, the basic idea is that the outer shell protects the doped UCNP core from unwanted quenching by the environment (e.g., quenching by OH-vibrations of water molecules). However, it has been shown that the shielding effect by this outer layer is smaller than expected. One of the limitations found is the intermixing of dopant ions from the different layers. This intermixing process has been demonstrated, e.g., by TEM investigations. Examples are given by Hudry et al. revealing an intermixing layer formed during the synthesis of core-shell nanostructures [10,11,12,13]. A recent work by Diogenis et al. contributes to these findings and reveals a major excitation of Eu(III) in the core-shell interfacial region by an energy transfer from Gd(III) [14]. Liu et al. observed Ln(III) migration already at low temperatures, as low as 200 °C, and showed a dependency on the Ln(III) concentration [15]. In good agreement to that, Chen et al. suggested increased Ln(III) migration at higher temperatures related to vacancies in the crystal lattice and higher vibrational energy of the dopants [16]. Dong et al. suppressed triple charged Ln(III) migration by growing a CaF_2_ shell with double charged Ca(II) ions [17], which is in good agreement with the Goldschmidt rules/tolerance factors [18].

The intermixing of Ln(III) ions has also been previously investigated with laser spectroscopy in our group. The monitoring concept is based on the lanthanide resonance energy transfer (LRET) [19]. Our previous work has focused on a NaNdF_4_/NaYF_4_ host lattice with a core-shell-shell structure. The first shell has been an insulation shell (also called insulation layer), being only composed of NaYF_4_, to create a variable spatial separation of LRET-donor and LRET-acceptor ions in the outer shell and the core, respectively [19]. The photoluminescence (PL) emission of Eu(III) ions (donor) has been analyzed within the resonance energy transfer framework to calculate the average number of acceptor ions (being Nd(III)) around one Eu(III) ion.

Based on the previous experiments, we have extended our research on the intermixing of dopant ions between core and shell in crystalline nanoparticles. An additional LRET-pair has been used in the NaYF_4_ host lattice. Since Nd(III) possesses only a weak luminescence in the visual spectral range, it was replaced by Pr(III). Pr(III) shows good PL emission in the visible spectral range and can be used as LRET-acceptor in combination with Eu(III) [20]. The change in the acceptor ion aims to record the acceptor (here: Pr(III)) PL emission as an additional parameter. The Pr(III) luminescence sensitization by Eu(III) is analyzed complementary to the Eu(III) PL emission, which is quenched. Additionally, the extent of intermixing behavior of Nd(III) and Pr(III) is discussed based on our previous findings with Nd(III) in the NaYF_4_ host lattice [19]. Second, the former investigated LRET-pair (Eu-Nd) will be transferred into a NaGdF_4_ host lattice (instead of NaYF_4_ as in Ref. [19]) (vide infra) and the effect of the lattice on the intermixing is addressed. NaGdF_4_ is expected to crystallize preferably in the hexagonal phase, because the NaYF_4_ lattice forms a cubic phase at low temperatures but tends strongly to form the desired hexagonal phase upon doping with Gd(III) ions [6,21,22,23]. Here, the comparison of Nd(III) in NaGdF_4_ and in NaYF_4_ (from the previous work) shall be realized. The hexagonal crystal phase of NaYF_4_ (and of NaGdF_4_) is known to possess higher upconversion (UC) efficiency than the cubic crystal phase relating to the lower phonon energy and lower crystal field symmetry of the hexagonal phase [6,21,24,25]. Additionally, the NaGdF_4_ host lattice equips the nanoparticles with magnetic properties which could be exploited in magnet resonance imaging or potentially in multidimensional diagnostic applications [24]. Therefore, the characterization of the intermixing in this particular phase is of special interest. In this work, we investigate the use of the two LRET-pairs Eu-Nd [20,25,26,27,28] and Eu-Pr [20,29,30,31] in different host lattices for the investigation of the migration of Ln(III).

## 2. Materials and Methods

### 2.1. Materials

All chemicals were used as received without previous purification. From Sigma Aldrich (St. Louis, MO, USA) were purchased: RECl_3_·6H_2_O (RE:Eu^3+^, Pr^3+^, Y^3+^, Yb^3+^, purity >99.99%) and ammonium fluoride (NH_4_F, ≥99.99%). From Alfa Aesar (Kandel, Germany) were purchased: RECl_3_·6H_2_O (RE: Gd^3+^, Nd^3+^, purity > 99.99%) and oleic acid (OA, 90%). From Carl Roth (Karlsruhe, Germany) were purchased: cyclohexane (ROTISOLV^®^ ≥ 99.9%), ethanol (≥99.8%, 1% MEK) and sodium oleate (NaOA, 90%). The solvent Therminol^®^ 66 was bought from FRAGOL GmbH + Co KG (Mülheim, Germany).

### 2.2. Concept of This Study

**Figure 1:**
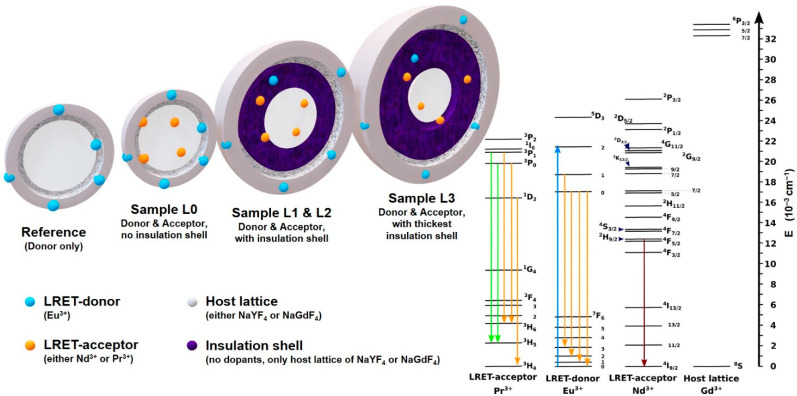
Illustration of the nanoparticle design and concept (**left**). Colored spheres are: Eu(III) in blue and either Nd(III) or Pr(III) in orange. Light and dark grey illustrates the host lattice of the cores and the shells being either NaYF_4_ or NaGdF_4_. The purple intermediate shell is the insulation shell and consists of the undoped host lattice material of the core. The acceptors Nd(III) or Pr(III) are doped in the core. The donor Eu(III) is doped in the outer shell. (**Right**) Illustration of the energy levels of Pr(III), Eu(III), Nd(III) as FRET/LRET pairs and Gd(III) as host lattice ion. The transitions for the respective Ln(III) ions are: Blue upward arrow for 465 nm absorption yields excited Eu(III) in the ^5^D_2_ state, Pr(III) in the ^3^P_0_/^3^P_1_ (^1^I_6_) state and Nd(III) in the ^4^G_11/2_ (^2^D_3/2_ or ^4^G_9/2_) [38]. Downward arrows indicate the respective Ln(III) luminescent transitions. Vide infra for corresponding emission spectra. Gd(III) on the right indicates its large energy gap and its indifference on the LRET for Eu(III) quenching.

In our current study, two main sets of nanoparticles were investigated. The nanoparticles were synthesized as core @ shell @ shell particles (=CSS). The notation is as follows:

- Set Y300: Core = NaYF_4_:Pr_20%_ @ Shell = NaYF_4_ @ Shell = NaYF_4_:Eu_5%_;
- Set Gd300: Core = NaGdF_4_:Nd_20%_ @ Shell = NaGdF_4_ @ Shell = NaGdF_4_:Eu_5%_;

The percentages are mol% referring to the trivalent ions in the nanocrystal. The first shell is referred to as insulation shell/layer and its thickness has been varied in order to have different distances between the core and the outer shell. The composition, nanoparticle size, and insulation shell thickness for each set is summarized in Table 1. Each set has its respective reference sample without the acceptor ions in the core (pure host lattice) and without an insulation shell, indicated as Ref CS. The Ref CS samples have only Eu(III) ions doped in the outer shell. The L0 CS samples have been synthesized the same way as the Ref CS sample except for the acceptor doping being 20 mol% in the core (and only 80 mol% of Y(III) or Gd(III)). L1 CSS, L2 CSS and L3 CSS are as L0 CS but with an additional intermediate shell (see Figure 1, inner purple shell), that has been grown prior to the donor doped outer shell. The additional intermediate shell increases in thickness, which is indicated by increasing numbers in the sample declaration. The insulation shell separates the donor and the acceptor spatially from each other. The sample L1 CSS is based on the sample L1 CS, which is derived from the core of L0 C. An overview of two main sets is given in Table 1. In Table 1, only the diameters of the nanoparticles with the first shell (so, with the insulation shell, except for L0 CS) is given, as this is the important information with respect to the distance between donor and acceptor for the application of the LRET model described below (Equations (1)–(3)). The notation for the samples L1 CS, L2 CS and L3 CS corresponds to the nanoparticles prior to the growth of the outer shell, which is doped with Eu(III). The described nanoparticle design is illustrated in Figure 1, in which the respective energy levels of the applied Ln(III) ions are shown as well [32,33,34,35,36,37]. In addition to the main sets, two subsets were synthesized in order to clarify certain effects (vide infra and Appendix A, Table A1). Briefly, the Y300_UCNP and the Gd200 subsets were synthesized according to the same protocol used in the other respective sets. The Y300-UCNP set is the same set as Y300 except for the core doping, which has been changed to the upconversion pair of Yb(III) with 18 mol% and Pr(III) with 2 mol%. With this set, the upconversion luminescence of Yb- (upconversion sensitizer) and Pr-ions (upconversion activator and LRET-acceptor) and the LRET of Eu-to-Pr was investigated. The Gd200 set differs from the Gd300 set only by the synthesis temperature used which was reduced by 100 °C.

### 2.3. Nanoparticle Synthesis

All syntheses were performed as previously described [19,39], whereas the amounts of the RE trivalent cations (here: Pr(III) and Y(III) or Nd(III) and Gd(III)) had been adjusted.

#### 2.3.1. Core Synthesis of NaREF_4_ (UCNP)

Depending on the sample (compare composition in Table 1) the RE chlorides (YCl_3_·6H_2_O; GdCl_3_·6H_2_O, respectively 1 mmol) were used for the reference cores or in combination with the optical active RE chlorides for the core samples (0.8 mmol of Y(III) or Gd(III) and 0.2 mmol of Nd(III) or Pr(III) or as UC pair: 0.02 mmol Pr(III) and 0.18 mmol of Yb(III)). The RE chlorides, OA (25.2 mmol, 8 mL, 7.12 g) and the solvent Therminol^®^ 66 (12 mL) were transferred into a 50-mL-three-necked-flask. The reaction mixture was evacuated for 10 min at room temperature with subsequent heating to 140 °C under vacuum (<10 mbar) and vigorous stirring. 140 °C were maintained for at least 45 min, so that a clear solution was obtained. The reaction flask was vented with argon to add NaOA (2.5 mmol) and NH_4_F (4 mmol). After re-evacuation the temperature was set to 80 °C and kept for 30 min until all salts had dissolved. The reaction flask was re-vented with argon and heated up to 320 °C (heat rate: 25 °C/min) and kept for 15 min. Finally, the temperature was decreased to 250 °C by air and then to approx. 60 °C by a water bath. The nanoparticles were precipitated by ethanol and centrifuged at 3100 g for 8 min. Further purification was performed by washing with ethanol and re-centrifugation for three times. The final precipitate was dispersed in cyclohexane (15 mL).

With respect to the nanoparticle synthesis and the changing dopants, the host lattice change from NaYF_4_ to NaGdF_4_ is expected to work as before, since NaGdF_4_ crystallizes in $P\bar{6}$ space group [40] as well as NaYF_4_ and NaNdF_4_ [21,41,42,43,44,45]. A more detailed discussion can be found in Ref. [19]. It should be kept in mind, that even if the synthesis conditions are constant, it cannot be guaranteed that all the lattices crystallize in the same space group which can lead to lattice variations [46]. The trivalent ion migration within the crystal host lattice becomes possible based on those variations and on lattice vacancies, elevated temperatures, dopant concentration, as well as the synthesis approach and the design of the core-shell(-shell) systems.

#### 2.3.2. Shell-Precursor Synthesis of NaREF_4_ and NaREF4:Eu

The NaREF_4_ insulation shell (first shell) was prepared either with YCl_3_·6H_2_O or with GdCl_3_·6H_2_O (2 mmol, respectively). The outer NaREF_4_:Eu shell doped with 5 mol% Eu(III) was prepared with the same RE chlorides as before (but: 1.9 mmol of the Y/Gd chlorides; 0.1 mmol EuCl_3_·6H_2_O). The respective RE chlorides were transferred together with OA (4 mL, 3.56 g) and Therminol^®^ 66 (8 mL) into a 50-mL-three-necked-flask. The flask was evacuated for 10 min, subsequently heated up to 140 °C and kept at this temperature for 45 min until a clear solution had formed. The reaction mixture was cooled down to 50 °C to add under an argon counter stream NaOA (2.5 mmol) and NH_4_F (4 mmol). After re-evacuation, the system was kept for at least 30 min at 80 °C until the salts had dissolved. The flask was vented with argon and the precursor was stored with an argon atmosphere.

#### 2.3.3. Core-Shell and Core-Shell-Shell Synthesis

The respective nanoparticle cores (60 mg) were transferred into a 50-mL-three-necked-flask and OA (8 mL, 7.12 g) and Therminol^®^ 66 (8 mL) were added. This mixture was evacuated for 30 min at 75 °C and then vented with argon. The temperature was increased to 305 °C as fast as possible and the precursor solution was added at a rate of 2 mL/h. The volume addition of the insulation shell precursor relates to the increasing shell thickness and sample number: L1 = 0.5 mL; L2 = 1 mL; L3 = 4 mL (for set Y300) and L1 = 0.4 mL; L2 = 2 mL; L3 = 4 mL (for set Gd300)—the volume of the Eu(III) doped shell precursors was 1 mL—these declarations apply for all sets. After the precursor addition was completed, the precursor addition temperature (305 °C) was maintained for 5 min. The reaction mixture was cooled down and purified as described for the core nanoparticles. The final precipitate was dispersed in cyclohexane (8 mL).

### 2.4. Luminescence Emission Spectroscopy

The PL spectra and decay curves were recorded using a wavelength tunable pulsed Nd:YAG/OPO laser system (10 Hz, 26 mJ per pulse/130 mW). A Quanta Ray laser from Spectra Physics (Mountain View, CA, USA) was used for the excitation of the OPO (optical parametric oscillator) from GWU-Lasertechnik Vertriebsges. mbH (Erftstadt, Germany). The experimental setup was in a 90° angle of excitation and emission light. The emitted photons were recorded with a Shamrock SR303i spectrograph from Andor Technology (Belfast, Great Britain). The spectrograph has a grating with 600 L/mm blazed at 500 nm and an iStar DH720-18V-73 intensified CCD-camera from Andor Technology. Luminescence decay curves were recorded using a stroboscopic technique [47]. The initial delays were set to 500 ns for static luminescence emission spectra and to 200 ns for recording luminescence decay curves. The delay was gradually increased by a linear time base function, so that smaller time steps in the beginning and larger time steps in the end of the decays were realized. The data analysis was made with MATLAB 2020b (The MathWorks, Inc., Natick, MA, USA) and with OriginPro 2020b (OriginLab Corporation, Northampton, MA, USA).

### 2.5. Size (TEM) and Structural (XRD) Characterization

Transmission electron microscopy (TEM) images were recorded with a Tecnai G2 F20 X-Twin TEM from FEI/Thermo Fisher Scientific being operated at 200 kV acceleration voltage. The images were evaluated and the nanoparticles sizes determined with help of the software ImageSP Viewer/Image Sys Prog (version 1.2.5.16 × 64).

The powder X-ray diffraction (XRD) patterns of the nanoparticles were investigated with a PANalytical Empyrean powder X-ray diffractometer in Bragg-Brentano geometry equipped with a PIXcel1D detector. The Cu Kα radiation (λ(Kα) = 1.5419 Å) was used with a voltage and current of 40 kV and 40 mA. The detector sensitivity level (PHD level) was adjusted to 45–80 to reduce fluorescence. The active length was set to 3.0061°. The theta-theta scans were performed over a 2θ range of 4–70° with a step size of 0.0131° and over 190 min.

### 2.6. Theory

The obtained PL time-resolved emission spectra are analyzed with a stretched exponential model, Equation (1) [48], and an equation derived from the Förster theory, that expresses the number of acceptors around one theoretical donor, Equations (2) and (3) [47]. This model is denoted as LRET model. The stretched exponential model has been chosen to account for the slight differences in the microenvironment of Eu(III). The stretched exponential model is a robust and simple approach to describe the spatial distribution of the Eu(III) in the host lattice with a small number of fitting parameters.

(1) $$I_{D}\left(t\right){=I}_{D}\left(0\right)\exp\left[-{\left(\frac{t}{\tau_{D}}\right)}^{\beta_{D}}\right]{+y}_{0}$$

The Index *D* stands for the donor in absence of the acceptor. *I_D_*(*t*) is the donor PL emission intensity to the given time *t*. Hence, *I_D_*(0) is the initial PL emission intensity and the amplitude for the model. *τ_D_* is the donor luminescence decay time and *β_D_* is a heterogeneity parameter describing the donor’s microenvironment and its tiny variations, in absence of acceptors, respectively. If *β_D_* > 1, the model will be a stretched exponential function which can be interpreted as a continuous distribution of PL decay times [48]. If *β_D_* = 1, the model will be a mono-exponential function indicating a homogeneous microenvironment for the emitting donors in the host lattice. *y*_0_ accounts for the background signal.

(2) $$I_{\mathrm{DA}}\left(t\right){=I}_{\mathrm{DA}}\left(0\right)\exp\left[-{\left(\frac{t}{\tau_{D}}\right)}^{\beta_{D}}\;-\;2\gamma \;{\left(\frac{t}{\tau_{D}}\right)}^{\alpha /2}\right]{+y}_{0}$$

(3) $$\gamma =\frac{\sqrt{\pi }}{2}c_{A}\frac{4}{3}{\pi \;R}_{0}^{3}\;$$

The index *DA* indicates the donor in presence of the acceptor, *D* as above the donor only. *I_DA_*(*t*) is the donor PL emission intensity to the given time *t*. Hence, *I_DA_*(0) is the initial luminescence emission intensity and amplitude of the mode. *y*_0_ accounts for the background signal. *τ_D_* and *β_D_* are adopted from the donor PL decay model of the respective reference sample with the absence of the acceptors. The additional heterogeneity *α* parameter has been introduced to account for the acceptor distribution and its related microenvironments. The parameter *γ* scales with the number of acceptors in a three-dimensional sphere, having a donor as center. The sphere has the radius of the Förster radius *R*_0_ of the respective donor-acceptor pair. Here, the acceptor concentration *c_A_* is given in ions per Å³. The term *c_A_*$\frac{4}{3}\pi R_{0}^{3}$ expresses the average acceptor number (number of ions) in this 3D sphere with radius equal to *R*_0_ around the donor.

For the sake of clarity, the described model will be called LRET model, the acceptor number will be denoted as acceptor concentration (or as “#acceptors”) and the applied donor-acceptor pairs are either Eu(III) and Nd(III) with *R*_0_ = 8.53 Å or Eu(III) and Pr(III) with *R*_0_ = 8.2 Å [20]. In case of Nd(III) the resonance condition is fulfilled, e.g., for the ^2^G_7/2_, ^4^G_5/2_ ← ^4^I_9/2_, ^2^H_11/2_ ← ^4^I_9/2_, and ^4^F_9/2_ ← ^4^I_9/2_ [28,38], while for the Pr(III) the resonance is achieved via the ^1^D_2_ ← ^3^H_4_ [30,38] (see Figure 1).

The LRET efficiency *E*_LRET_ is calculated with Equation (4) based on the donor PL decay time in presence (*τ_DA_*) and in absence of the acceptor (*τ_D_*). The parameter $\tau_{D}$ had been calculated before with Equation (1). The parameter $\tau_{\mathrm{DA}}$ had been calculated with Equation (1) as well, but here $\tau_{D}$ (and $\beta_{D}$) was replaced by $\tau_{\mathrm{DA}}$ (and $\beta_{\mathrm{DA}}$), which parameters are also listed in Table 2 and Table 3 and in the Appendix A, Table A2, Table A3 and Table A4. Equation (1) would then look like:

$$I_{\mathrm{DA}}\left(t\right){=I}_{\mathrm{DA}}\left(0\right)\exp\left[-{\left(\frac{t}{\tau_{\mathrm{DA}}}\right)}^{\beta_{\mathrm{DA}}}\right]{+y}_{0}\;$$

(4) $$E_{\mathrm{LRET}}=1\;-\;\frac{\tau_{\mathrm{DA}}}{\tau_{D}}\;$$

Because of the Pr(III) PL decay time being shorter than the Eu(III) PL decay time [19,49,50,51,52], the PL decay curves of Pr(III) and Eu(III) were obtained by deconvolution of the respective experimental decay kinetics using parallel factor analysis (PARAFAC algorithm of MATLAB [53]), where necessary. Constrains were set to avoid negative values in the time base, wavelength and intensity. The deconvoluted decays were fitted using Equations (1) and (2) (with OriginPro) for the donor PL decay times and the acceptor PL decay times listed in the results section.

The presented acceptor PL decay times (in absence of the donor, indicated as “*A*” for CS samples, and in presence of the donor, indicated as “*AD*” for CSS samples) were also calculated with a stretched exponential decay model which transforms Equation (1) into:

$$I_{A\;(\mathrm{AD})}\left(t\right){=I}_{A\;(\mathrm{AD})}\left(0\right)\exp\left[-{\left(\frac{t}{\tau_{A\;(\mathrm{AD})}}\right)}^{\beta_{A\;(\mathrm{AD})}}\right]{+y}_{0}$$

## 3. Results

### 3.1. Structural Characterization

**Figure 2:**
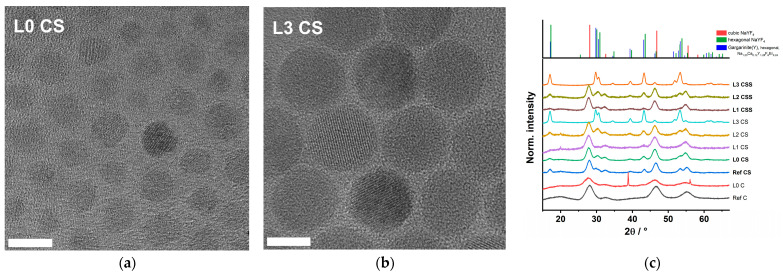
Set Y300: TEM images of (**a**) L0 CS, (**b**) L3 CS and (**c**) XRD data of NaYF_4_:Pr_20%_ @ NaYF_4_ @ NaYF_4_:Eu_5%_. TEM images show the desired nanoparticle size increase upon shell growth. The common core L0 C is not shown for this set. An overview of all recorded TEM images is given in the Appendix A, Figure A1. The XRD data reveals the nanoparticles’ hexagonal phase. The top XRD trace shows the reference diffraction patterns of cubic NaYF_4_ (red, ICDD PDF #77-2042), hexagonal-NaYF_4_ (green, ICDD PDF #16-334), and hexagonal Gagarinite-(Y) (blue, ICSD #39696). The sharp diffraction peaks at 39° and 56° of L0 C are attributed to cubic NaF (ICSD #43611, reference not shown). Scale bar = 10 nm.

**Figure A1:**
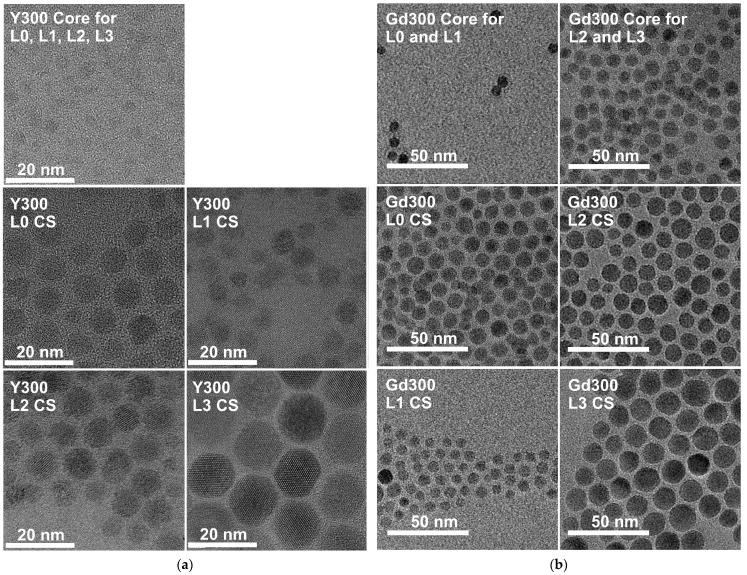
TEM images of the measured samples for the set (**a**) Y300 (NaYF_4_:Pr_20%_ @ NaYF_4_ @ NaYF_4_:Eu_5%_, with Pr(III) doping in the core and the insulation layer thicknesses) and (**b**) Gd300 (NaGdF_4_:Nd_20%_ @ NaGdF_4_ @ NaGdF_4_:Eu_5%_, with Nd(III) doping in the core and the insulation layer thicknesses). The step of the insulation shell growth synthesis results in increasing particles in comparison to the initial core particle diameter. The NaYF_4_ based nanoparticles show a larger size distribution of particles than the NaGdF_4_ nanoparticles.

Two representative examples of TEM images of set Y300 (being the NaYF_4_:Pr_20%_ @ NaYF_4_ @ NaYF_4_:Eu_5%_ nanoparticles synthesized at 320/305 °C) are shown in Figure 2 (other TEM images are shown in the Appendix A, Figure A1). As expected, L3 CS has a larger diameter than L0 CS, since L0 CS has been prepared with 1 mL of Eu-doped precursor solution and L3 CS with 4 mL of the insulation shell precursor solution leading to the larger shell thickness. The TEM images of set Gd300 (NaGdF_4_:Nd_20%_ @ NaGdF_4_ @ NaGdF_4_:Eu_5%_) nanoparticles synthesized at 320/305 °C are shown in the Appendix A, Figure A1. In Table 1, the nanoparticle sizes of intermediate step, the CS samples, and their respective insulation shell thicknesses are summarized.

**Figure A2:**
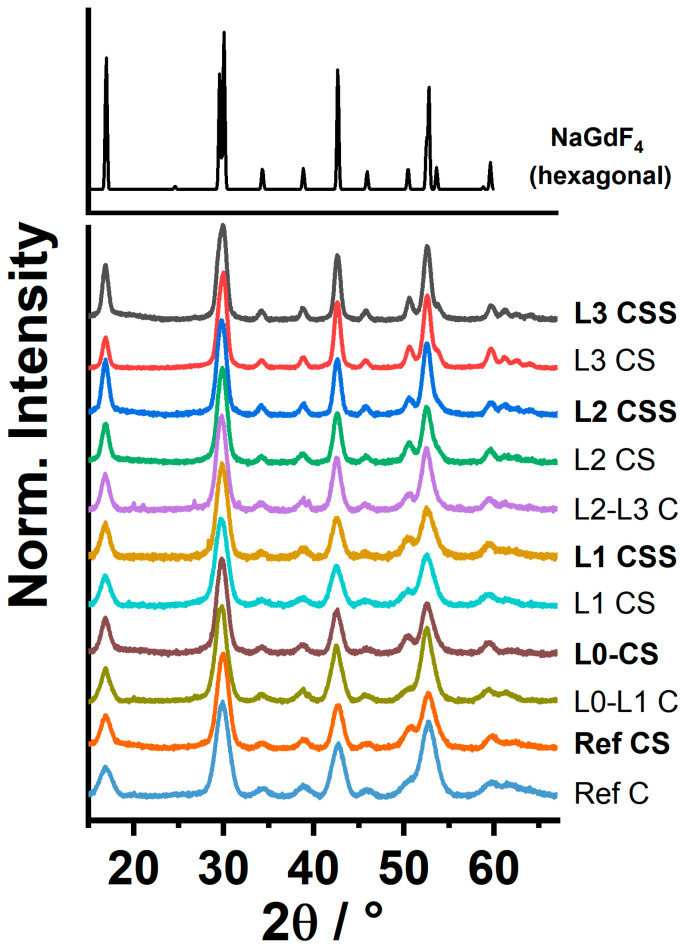
Set Gd300: Powder X-ray diffraction (XRD) patterns (NaGdF_4_:Nd_20%_ @ NaGdF_4_ @ NaGdF4:Eu_5%_). The XRD reference patterns on top = hexagonal NaGdF_4_ (ICSD: 415868). The diffraction patterns of the samples match well with the reference patterns revealing their hexagonal crystal phases.

The XRD investigations reveal good agreement between the reference XRD patterns and the patterns of the synthesized NaYF_4_ (Figure 2c) and NaGdF_4_ (Appendix A, Figure A2) nanoparticles. Some samples of the NaYF_4_ samples (set Y300) show reflexes of the cubic NaYF_4_ which vanish gradually after shell addition (see Figure 2c). Furthermore, the XRD patterns of the core nanoparticles (of set Y300) show sharp reflexes at 39° and at 56° corresponding to NaF.

### 3.2. Luminescence of Set Y300

Compared to our previously published work in the set Y300 the acceptor Nd(III) was exchanged for Pr(III), which is only slightly larger than the former but has the advantage to show luminescence in the visible spectral range. Since it can also serve as an acceptor in combination with Eu(III) as donor, its luminescence may also be used to gain complementary information with respect to the sensitization due to LRET.

**Figure 3:**
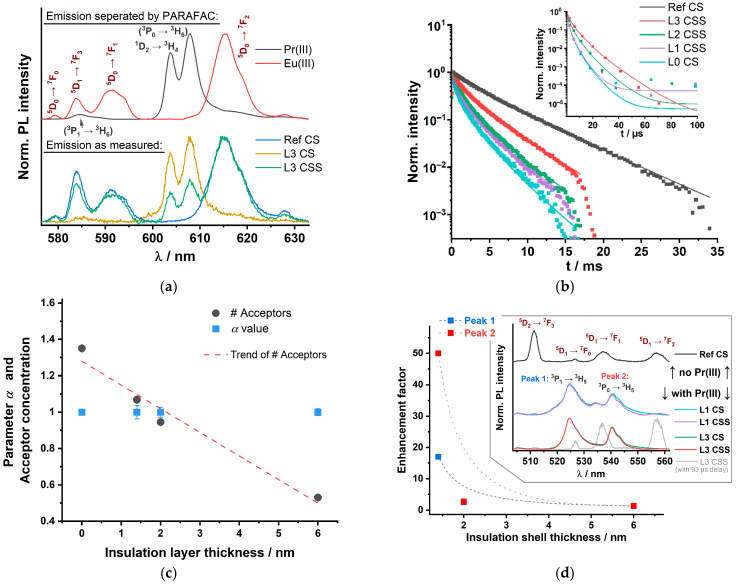
Set Y300 (NaYF_4_:Pr_20%_ @ NaYF_4_ @ NaYF_4_:Eu_5%_): Spectroscopic investigation of the Eu(III) and the Pr(III) emission of CSS nanoparticles. (**a**) Eu(III) and Pr(III) PL emission spectra around 600 nm (red labels = Eu(III) transitions, black labels = Pr(III) transitions, λ_ex_ = 465 nm). The Pr(III) transitions labeled in brackets may result from direct excitation as well as sensitization of the ^3^P_i_ ← ^3^H_6_ transition by the ^5^D_0_ state of Eu(III). Ref CS has no Pr(III) (no acceptor in the core, Eu(III) (donor) in the shell). L3 CS contains Pr(III) (acceptor) in the core and is equipped with the insulation shell, so no Eu(III) (donor) in the shell. L3 CSS contains both ions, Pr(III) (acceptor) in core, no doping in the insulation shell, and Eu(III) (donor) in the outer shell. The PARAFAC separated emission spectra (top part) were calculated from L3 CSS raw PL emission data. (**b**) Eu(III) luminescence decay kinetics recorded at λ_em_ = 616 nm (corresponds to the ^5^D_0_ → ^7^F_2_ transition of Eu(III), λ_ex_ = 465 nm). With decreasing insulation shell thickness, the Eu(III) PL decay times decrease. In addition, from a visual inspection, it can be seen that the kinetics are no longer following a monoexponential decay as shown by Ref CS. Inset of (**b**) are the Pr(III) PL decay curves for λ_em_ = 608 nm (^1^D_2_ → ^3^H_4_) being separated by PARAFAC from the Eu(III) PL emission at 616 nm (λ_ex_ = 465 nm). (**c**) Results of the evaluation of Eu(III) kinetics based on Equation (2): average acceptor concentration within a 3D sphere (radius of $R_{0}$(Eu/Pr) = 8.2 Å) and parameters *α*, in dependence on the insulation shell thickness, respectively. With increasing insulation shell thickness, the average acceptor numbers decrease. Parameters *α* are not affected by the thickness of the insulation shell. Detailed regression parameters are shown the Appendix A, Table A2. (**d**) Enhancement factors (*τ*_(CSS)_/*τ*_(CS)_) of the Pr(III) PL decay times at 524 nm and at 540 nm (potentially resulting from direct excitation as well as sensitization by Eu(III) re-populating the ^3^P_1_ and ^3^P_0_ state, as in (a)). Inset of (**d**): PL emission with its respective transitions of Pr(III) in black and Eu(III) in red around 530 nm with λ_ex_ = 465 nm. Detailed regression parameters of the Pr(III) PL decay curves are in the Appendix A, Table A3.

In Figure 3a, examples for the luminescence spectra of the Y300 nanoparticle set are shown (bottom part). After excitation at λ_ex_ = 465 nm, the recorded emission spectra contained contributions of Eu(III) as well as of Pr(III) luminescence. The observed Pr(III) luminescence is a result of sensitized and direct excitation. The direct sensitization occurs via the ^1^D_2_ ← ^3^H_4_ of Pr(III). The other Pr(III) luminescence bands (see Figure 1, transition in brackets, and inset of Figure 3d) observed may be a combination of direct excitation (into ^3^P_1_), relaxation (e.g., into ^3^H_5_ or ^3^H_6_) and a (possible) subsequent sensitization (to ^3^P_0_ and ^3^P_1_). That a sensitization occurs can be seen from the differences in the decay times found for the nanoparticles without and with Eu(III) (see Table A3 containing the PL decay and enhancement data for the transitions ^3^P_1_ → ^3^H_5_ and ^3^P_0_ → ^3^H_5_ at 524 nm and 540 nm, respectively). We used PARAFAC to calculate the pure Eu(III) and the pure Pr(III) luminescence spectra (see Figure 3a, top part). Because of the fact that the Eu(III) PL emission also contains a fairly high contribution of luminescence arising from the ^5^D_1_ → ^7^F_3_ transition (around λ_em_ = 585 nm) the luminescence kinetics were evaluated for the ^5^D_0_ → ^7^F_2_ transition at λ_em_ ≈ 616 nm. Although emission bands of Pr(III) are also spectrally close (Pr(III) also has transitions in that spectral range: ^3^P_1_ → ^3^H_6_ at 585 nm as well as ^1^D_2_ → ^3^H_4_ and ^3^P_0_ → ^3^H_6_ at 608 nm [38,54]), the Pr(III) PL decay kinetics are much faster (vide infra) and therefore, the Eu(III) emission decay kinetics can be evaluated selectively. The luminescence decay kinetics of Eu(III) and Pr(III) are shown in Figure 3b. It can be seen that upon decreasing the thickness of the insulation layer the Eu(III) luminescence decay kinetics became faster. The observed decrease can be attributed to the LRET process between Eu(III) and Pr(III). It is intriguing that a distinct change is also found in the L3 CSS PL decay kinetics (see Figure 3b), although the thickness of the insulation layer was more than 7-times the Förster distance (insulation layer thickness = (6.0 ± 0.5) nm compared to *R*_0_(Eu/Pr) = 0.82 nm) (also see Table 2). Hence, mixing of Pr(III) and Eu(III) ions during synthesis into the insulation layer occurred, subsequently the average distance between donor and acceptor ions became much smaller than the insulation shell thickness, which makes the LRET possible. The inset of Figure 3b shows the Pr(III) PL decay times, that result from the PARAFAC analyzed decay curves and spectra of the emission at 608 nm. In Figure 3c the dependence of the average acceptor number on the insulation layer thickness is shown. In addition, the parameter *α* is shown, which is not changing with the insulation layer thickness basically indicating that there is no insulation layer related heterogeneity of the acceptor distribution. The results found for the Eu(III)/Pr(III) pair in the NaYF_4_ host lattice are in very good agreement with our results reported for Nd(III) as the acceptor ion.

In addition to the Eu(III) emission also the luminescence of Pr(III) was investigated. Since the decay kinetics of Pr(III) luminescence are much faster than that of Eu(III), we were expecting to find an increased acceptor luminescence decay time due to LRET. However, we observed the contrary: decreasing luminescence decay time with decreasing insulation layer thickness (see inset of Figure 3b and Table 2, detailed regression parameters in the Appendix A, Table A2). In order to find an explanation for the observed trend, the Lx CS (x = 1–3) samples were investigated, in which no outer Eu(III) containing shell was present. The Lx CS samples were used as a reference for “no LRET”. Interestingly, when comparing the luminescence decay times of the Lx CS with its respective Lx CSS sample (x = 1–3, see Table 2), we found that the τ-values for the Lx CSS samples were always larger. The enhancements (ratio τ-value for Lx CSS/Lx CS) are given in Table 2. The largest enhancement was found for L1 CSS, which had the thinnest insulation layer. We interpret this observation as the result of two opposing effects. High concentration of Pr(III) in the core leads to self-quenching, but due to the intermixing with the shell, the concentration in the core and subsequently the self-quenching is reduced. The extent of concentration reduction in the core is dependent on the thickness of the shell (insulation layer). Therefore, it is smallest for L1 and largest for L3. On the other hand, the LRET should be largest for L0 and L1, but smallest for L3. From our data, it may be concluded that the dilution effect is dominating. But, by using the comparison with Lx CS samples, it is possible to show the LRET effect on the acceptor luminescence. The luminescence of Pr(III) was also investigated at additional emission wavelengths, for which the enhancement factors have been plotted (see Figure 3d). In the appendix the Pr(III) decay times *τ* of Peak 1 at 524 nm (^3^P_1_ → ^3^H_5_) and Peak 2 at 540 nm (^3^P_0_ → ^3^H_5_) are summarized (see Appendix A, Table A3). Here, basically the same trends were found supporting our findings.

### 3.3. Luminescence of Set Gd300

**Figure 4:**
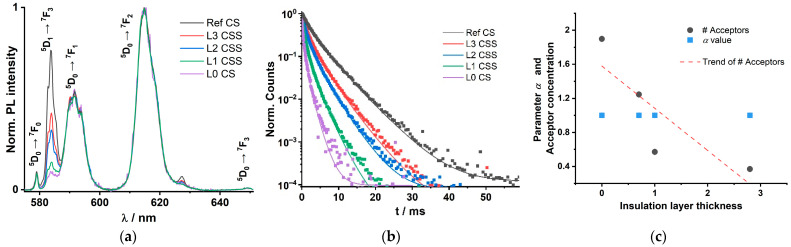
Set Gd300: Spectroscopic investigation of the Eu(III) emission of CSS nanoparticles (NaGdF_4_:Nd_20%_ @ NaGdF_4_ @ NaGdF_4_:Eu_5%_). (**a**) Normalized (by maximum) Eu(III) emission spectra of set Gd300 (λ_ex_ = 465 nm). (**b**) Eu(III) luminescence decay kinetics, emission measured at 616 nm (^5^D_0_ → ^7^F_2_) (λ_ex_ = 465 nm). With decreasing insulation shell thickness, the Eu(III) decay time decreases because of decreasing Eu(III)-Nd(III) distance. (**c**) Graphical presentation of the acceptor concentration within a 3D sphere with the radius of *R*_0_(Eu/Nd) = 8.53 Å and parameters *α*, in dependence on the insulation shell thickness, from the evaluation of Eu(III) kinetics with the LRET model equation (Equations (1) and (2)). With increasing insulation shell thickness, the acceptor numbers decrease, whereas the parameters *α* result constantly at the value one.

The Eu(III) emission spectra of the set Gd300 being quenched by the Nd(III) (LRET-acceptor) are shown in Figure 4 (λ_ex_ = 465 nm). In Figure 4a, the Eu(III) PL emission spectra of set Gd300 are shown with the respective assignment of electronic state transitions. The spectra were normalized to the maximum of the ^5^D_0_ → ^7^F_2_ transition. In Figure 4b, the Eu(III) PL decay kinetics are shown indicating decreasing luminescence decay times (judged by the increasing slope of the decay curves, see Table 3) with decreasing insulation shell thickness. The reference sample (no Nd(III) in the core) has the longest decay time (see Table 3). Even for the sample L3 CSS having the largest insulation shell thickness of 2.8 nm (exceeding the Förster radius *R*_0_(Eu/Nd) = 0.853 nm by a factor >3) a distinct quenching is found.

Based on the LRET-model, the decreasing Eu(III) PL decay times translate in increasing acceptor concentrations as the insulation shell thickness decreases. The Gd300 sample Ref CS has an initial decay time of 2814 µs (λ_ex_ = 465 nm). The PL decay time decreases from L3 CSS to L0 CS from 1505 µs down to 233 µs. Therefore, the LRET efficiency and subsequently the calculated average acceptor concentrations increase from 0.4 acceptors in the 3D sphere (according to the LRET model) for L3 CSS up to 1.9 for L0 CS (see Table 3 and Figure 4c). Looking at the heterogeneity parameters *α* and *β*, no significant alterations in the microenvironments of the donor or the acceptor ions are indicated.

A striking difference between the two lattices investigated is the intensity of the ^5^D_1_ → ^7^F_3_ transition, which is visible in both NP sets. The strong contribution of this transition to the overall detected emission is unusual. Comparing the Y300 set with the Gd300 set, the ^5^D_1_ → ^7^F_3_ transition is (i) more intense (judged by a comparison with the intensity of the ^5^D_0_ → ^7^F_j_ transitions) and (ii) it seems to be more affected by the presence of Nd(III) (compare Figure 3a and Figure 4a). Based on the latter observation, it is tempting to assume a participation of the Eu(III) ^5^D_1_ energy level in the LRET process.

**Figure 5:**
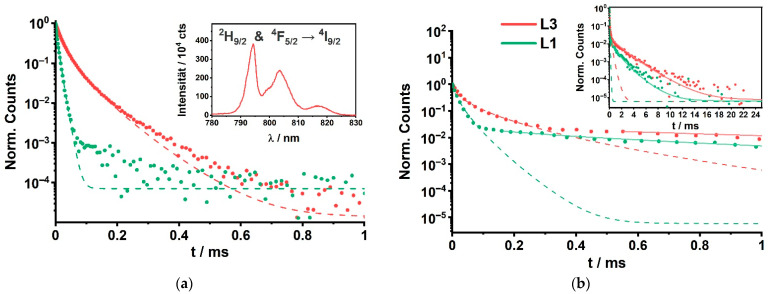
Set Gd300: Nd(III) PL decay curves of (**a**) CS and (**b**) CSS nanoparticles (λ_ex_ = 465 nm). Dotted curves are the experimental PL decay curves; dashed curves are the regressions of the Nd(III) PL decay; solid curves are the regressions of Nd(III) PL decay sensitized by Eu(III), regressions performed with Equation (1). (**a**) Nd(III) PL decay curves of the CS samples (inset: emission spectrum of L3 CS, representative for all recorded Nd(III) spectra of the Gd300 set). (**b**) Nd(III) PL decay kinetics of CSS samples within the first millisecond after excitation (Inset: full Nd(III) luminescence decay kinetics). Parameters listed in Table 4.

Complementary to the investigation of the acceptor-related luminescence of the Y300 set doped with Pr(III), the Nd(III) luminescence around 800 nm was analyzed for the Gd300 set. In Figure 5 the luminescence decay kinetics of the respective CS and CSS samples for the smallest and largest insulation layer (L1, L3, respectively) are shown. Alike in the case of the Y300 set, the acceptor decay kinetics were influenced by the thickness of the insulation layer (see Figure 5a). An increasing insulation layer thickness yielded also a decrease in the luminescence rate constant. That is in line with our interpretation of a partly dilution effect due to the intermixing process leading to a reduced concentration quenching of the Nd(III) ions in the core. In Figure 5b the luminescence decay kinetics of the corresponding CSS samples are shown.

The decrease of the luminescence decay rate due to the insulation layer related dilution of the Nd(III) ion in the core is observed. However, for the CSS samples a second much slower luminescence decay process is found (see inset of Figure 5b). This can be attributed to the LRET process and the resulting decrease (increase) of the luminescence decay rate (time). However, it does not become stronger with decreasing insulation layer thickness, because the concentration related quenching seems to be larger than the Nd(III) sensitization by Eu(III)-LRET (compare inset Figure 5b and values in Table 4 in which L1 (thin insulation shell) decays faster than L3 (thick insulation shell)).

## 4. Discussion

### 4.1. Structural Characterization

The XRD experiments reveal a hexagonal crystal phase for both sets (Y300 and Gd300), because of the good match between the samples XRD reflexes and the hexagonal reference XRD reflexes. For the set Y300, the detected reflexes of the cubic NaYF_4_ phase vanish with longer reaction time. Especially, L0 C and Ref C indicate purely cubic phased NaYF_4_ nanoparticles. Related to the findings of the TEM investigation two ideas came up: Firstly, the cubic phased nanoparticles could have either transformed into the desired hexagonal phased nanoparticles related to the respective precursor shell additions and the associated longer reaction times, or the precursor materials have grown themselves in a hexagonal phase on the cubic phased cores. Previous research by Voss and Haase, as well as Rinkel et al. and Dong et al. give examples for that. Voss and Haase and Rinkel et al. dealt with the fabrication of hexagonal phased UCNPs by providing cubic phased UCNPs as sacrificial material yielding in a narrow size distribution of hexagonal phased UCNPs which is majorly based on Ostwald ripening [55,56,57]. However, the TEM investigations do not support the dissolution of the initially formed cubic phased nanoparticles. A later work by Rinkel et al. reveals a conversion of the cubic to hexagonal phase NaYF_4_ particles [58], which could support the first idea. On the other hand, Dong et al. revealed by increasing the dosage of their Ca(II) precursors for growing the CaF_2_ shell on hexagonal phased UCNPs, that the XRD reflexes changed from hexagonal NaGdF_4_ towards CaF_2_ [17], which might indicate in the case here, a certain dependency of the XRD signal on the thickness of the shell (and the associated longer reaction time). In the case for these presented experiments, with the insulation layer of the same host material but increasing shell thicknesses, it could point towards the second idea. Unfortunately, the TEM images (discussed in the following paragraph) do not reveal a difference of the cores and the shells due to the same host lattice. The dopant concentration does not seem large enough to reveal significant contrast differences in TEM. Nevertheless, it can be summarized, the samples L1 CS, L2 CS, L1 CSS, L2 CSS and Ref CS and L0 CS show a mixture of reflexes from cubic and hexagonal NaYF_4_. The samples L5 CS and L5 CSS show only hexagonal NaYF_4_ reflexes. We attribute this to a cubic-to-hexagonal transition, which already indicates the migration of the ions within the nanocrystal. This migration is surely not only limited to the Ln(III) ions.

The observed NaF XRD reflexes in Figure 2 relate to NaF, from which already small amounts are sufficient to provoke sharp reflexes in the XRD patterns. The NaF vanishes after the shell growth synthesis, which indicates either its consumption and integration into the nanoparticle during the reaction or its removal by the washing and centrifugation steps after the reaction.

The TEM investigation confirms increasing particle size upon shell material addition and subsequently the successful variation of the insulation layer thickness, which is the basis for the LRET analysis. Whereas, the differentiation of the core and shell structures was not possible. Here, two sets with different host lattices were synthesized at T = 320 °C for the core and at T = 305 °C for the shell growth reactions, see Figure 2 and Figure A1 (Appendix A) and Table 1. The synthesis approach, with its applied synthesis conditions, yields spherical shaped nanoparticles, which can be seen here in the TEM images (Figure 2 and Figure A1) and in Ref. [19].

However, it has to be kept in mind, that the changing sizes affect the luminescence properties of upconversion nanoparticles. Hence, it is very likely the same case for the nanoparticles investigated here. Although, some samples share the same core (from the same synthesis batch), their luminescence properties differ slightly as their size and shell thicknesses (insulation layer as well as donor-doped outer shell) change. An important point to note is that the largest nanoparticle (L3 CSS) possesses the thinnest outer shell and the largest surface, which may lead to stronger Eu(III) luminescence quenching.

### 4.2. LRET

**Figure 6:**
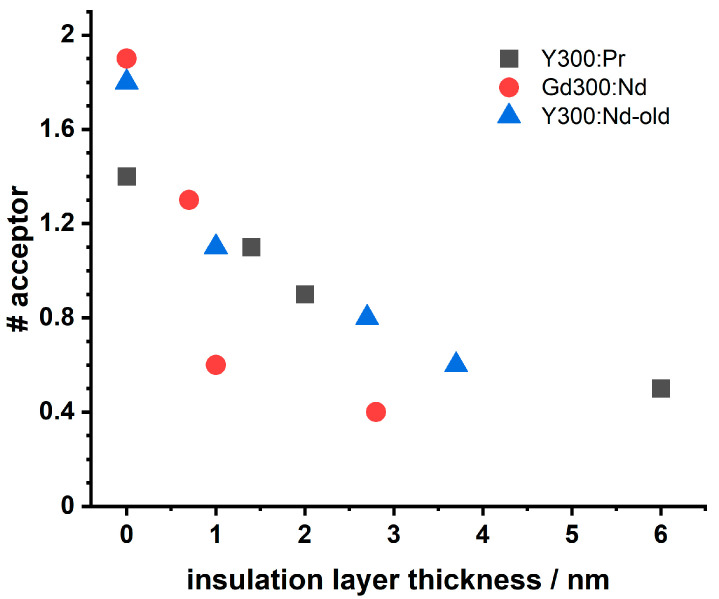
Comparison of the average acceptor number (#acceptor) in the respective *R*_0_ volume for the sets Y300 and Gd300. Shown are also data of Ref. [19] (Y300:Nd-old). The analysis is based on the donor PL emission data.

First, we analyzed the Eu(III) luminescence (donor) with respect to the LRET formalism, which was straight forward since any possible interferences from acceptor emission were discriminated by combining spectral and kinetic aspects in a PARAFAC analysis (vide supra, Figure 3). The analysis of the Eu(III) decay kinetics based on Equation (1) indicates, that the chemical environment does not change distinctly, since the heterogeneity parameter *β* decreases only slightly with decreasing insulation shell thickness (compare Table A2). This observation could be attributed to the comparable chemical behavior of the Ln(III) ions and the major presence of Y(III) ions in the NaYF_4_ host lattice in the core as well as in the shells. The same can be observed in the NaGdF_4_ host lattice with Gd(III) ions as a major lattice part. This is further supported by the heterogeneity parameter *α*, which represents the situation for the acceptor ions (vide infra). Based on Equation (2), also no change in *α* was found (compare Appendix A, Table A2 and Table A4 for Pr(III) and Nd(III), respectively). Therefore, within the used model the observed changes in the Eu(III) luminescence decay kinetics for the different insulation layer thicknesses are attributed to an alteration of the LRET efficiency. The insulation layer thickness has a clear effect on the Eu(III) luminescence decay kinetics: the luminescence decay time increased with increasing thickness. This was found for both acceptor ions (Pr(III) as well as Nd(III)) in the respective host lattices. This distance-dependent luminescence quenching was analyzed based on the LRET formalism (see Equations (1)–(4)). The LRET from Eu(III) to Pr(III) (or Nd(III)) cannot be suppressed—even if the insulation shell thickness exceeds the Förster radius *R*_0_ by a factor >7 (*R*_0_(Eu/Pr) = 8.2 Å and *R*_0_(Eu/Nd) = 8.53 Å, respectively). The average acceptor concentration (#acceptors) within the 3D sphere with the radius of *R*_0_ (of the respective LRET-pair) increases with decreasing insulation shell thickness (see Figure 6).

In Figure 6 the #acceptor (number of acceptors) for the different host lattices and different acceptor ions are compared. Data, resulting of research from Ref. [19], are shown as well. Although the data base is small (e.g, missing of reliable errors) some trends might be seen: (i) for the same host lattice (NaYF_4_) no difference in the intermixing of Pr(III) and Nd(III) are found and (ii) for the same acceptor ion (Nd(III)) a small influence of the host lattice is seen. It seems that in case of the NaGdF_4_ lattice the intermixing is less for the larger insulation thicknesses. For the latter observation, differences in the lattice constants as well as lattice phase in combination with specific acceptor ion properties (e.g., ionic radius) could be the reason. Here, small differences in the heterogeneity factors found for the NaYF_4_ and NaGdF_4_ lattices obtained from the LRET analysis might point in this direction (vide infra, Table A2 and Table A4). Furthermore, the ionic radii of Y(III) (121.5 pm), Gd(III) (124.7 pm), Eu(III) (126 pm), Nd(III) (130.3 pm), and Pr(III) (131.9 pm) [59] are within a deviation range of 15%, which should be noted. According to Goldschmidt’s theory these deviations are tolerable in terms of isomorphism for crystals [18,60]. However, the cationic radius of Y(III) deviates stronger from the Ln(III) cations. The apparent reduced intermixing of dopant Ln(III) ions in the NaGdF_4_ host lattice could correlate with a stronger migration competition between dopant Ln(III) ions and the host lattice Gd(III) ions, that reduces stronger the dopant Ln(III) ion migration than the Y(III) ions, being smaller, in the NaYF_4_ host lattice.

In addition to the analysis of the Eu(III) luminescence, we also analyzed the emission of the acceptor ions (Pr(III) for set Y300 and Nd(III) for set Gd300) in order to collect acceptor-based LRET data complementary to the donor results. The two donor-acceptor pairs Eu(III)/Nd(III) and Eu(III)/Pr(III) have been used before in LRET experiments [30,31,38]. However, in contrast to the analysis of the donor emission related data, for the acceptor luminescence some limitations are found with respect to selectivity in excitation and due to self-quenching because of the high local concentration of the respective acceptor ions in the core. In principle, for both acceptor ions a sensitization of their luminescence is shown. In order to quantify the sensitization effect induced by LRET (and separate contributions of direct acceptor excitation as well as self-quenching), the comparison between the CS and CSS samples of each set was necessary. Based on the data of the CS sets, it could be shown that a concentration of 20 mol% of the respective acceptor ion in the core is already high enough to induce concentration related self-quenching. The growth of an insulation layer is reducing the extent of self-quenching because acceptor ions from the core are intermixing with the shell (which is also an indication for the intermixing between core and shell). Here, this dilution effect becomes larger with increasing insulation layer thickness. Contrary to the self-quenching is the LRET based sensitization, which is largest for the L1 CSS samples with a thin insulation layer (see Table 2 and Table 4). Especially for the Nd(III) luminescence, the opposing trends (self-quenching vs. sensitization) are seen in its decay kinetics, in which two components were resolved (see Figure 5b). One was attributed to Nd(III) ion in the core, which suffer from self-quenching and the other to Nd(III) ions, which were mixed into the insulation layer. For the latter, the self-quenching was reduced and in case of an outer Eu(III) containing shell (CSS samples) the sensitization was effective. The LRET-based enhancement can be quantified by the comparison between the respective CS and CSS samples, (see Table 2, last row for Pr(III) as the acceptor ion and Table 4, last column for Nd(III), respectively).

## 5. Conclusions

The work presented is a sequel to our investigation of core-shell UCNPs and the intermixing of ions between core and shell during the synthesis. In continuation of our previous work, we have varied the host lattice composition as well as the Ln(III) ion used as acceptor in the core. For the chosen donor/acceptor pairs the donor (Eu(III)) luminescence can be detected without interference of the acceptor-related emission. Here, we also investigated the acceptor-related luminescence in order to monitor the intermixing between core and shell. In addition to the spectral discrimination between luminescence signals from donor and acceptor, in case of Pr(III) a time gating can be used additionally, since the respective luminescence decay time of Pr(III) is much smaller. In combination with chemometry (PARAFAC) the selective detection of the acceptor’s luminescence signal can be achieved. However, despite the advantages on the selective detection of the acceptor emission, we encountered a couple of draw backs in using the luminescence of Pr(III) or Nd(III) directly in the LRET analysis. Since the acceptor concentration in the core was high, we found a self-quenching, which was reduced upon adding a shell. With increasing shell thickness, the self-quenching was reduced indicated by the reference measurements using CS nanoparticles (no outer shell with Eu(III)), for which an increase in the acceptor luminescence decay time was found. This trend is opposite to the sensitization, for which also an increase in the acceptor’s luminescence decay time is expected (e.g., donor τ_Eu_ >> acceptor τ_Pr_), however here the largest sensitization is expected for the smallest insulation layer thickness. The effect of self-quenching is also of relevance for the standard composition of UCNP containing approx. 18 mol% of Yb(III) ions as sensitizer. Maybe by determining the luminescence kinetics of Yb(III) directly, the intermixing between core and shell can also be monitored, which then would be a potential “quick check” without synthesizing nanoparticles with tailored donor-acceptor pairs for LRET analysis. We will pursuit this idea in future experiments.

Based on the evaluation of the donor PL emission using the LRET concept an average number of acceptor ions in the Förster volume around the donor ions is determined and the dependence on the insulation layer thickness is found. For the first time, we also present luminescence data of the respective acceptor (Pr(III) or Nd(III)) and how it is influenced by the intermixing. Here, two trends of opposite directions are reported: (i) reduction of concentration related self-quenching due to mixing of the acceptor ions from the core into the insulation layer and (ii) sensitization due to LRET. In order to quantify the sensitization, it is necessary to differentiate between both effects and a reference sample set is needed. Therefore, the LRET data analysis of the donor emission is preferred because here no additional samples are needed.

For the purpose of building highly protective shell structures for UCNPs, the intermixing between protective shell(s) and the sublayers has to be minimized. Here, we tested a couple of synthesis and composition parameters with respect to their influence on the intermixing. Using Pr(III) or Nd(III) as acceptor ions in the core of NaYF_4_-based UCNP made no difference on the observed intermixing. Here, probably the difference in the ionic radii of Pr(III) and Nd(III) is too small to come into play. After all, the LRET approach is limited to certain donor acceptor pair combinations. On the other hand, the comparison of different host lattices from our data shows that the intermixing for the NaGdF_4_ lattice is smaller for medium and large insulation layer thicknesses (see Figure 6). It is tempting to attribute the observed effect to the difference in the matching between lattice cations (either Y(III) or Gd(III)) and dopant Ln(III) ions.

Here, work is in progress to investigate this parameter further. This is important because in the composition of UCNPs the “heavier” Ln(III) ions are normally used as sensitizer and activator. We have tested NP with a regular composition for UCNP in the core using our LRET approach (core doping 2 mol% Pr(III) and 18 mol% Yb(III), these are an activator and sensitizer pair for upconversion, and outer shell doping with Eu(III) whereas the insulation layer has been applied as before). However, the 2 mol% Pr(III) (activator and LRET-acceptor) were too small to induce a significant quenching of the Eu(III) luminescence (located in the outer layer of the CSS NP) and the Yb(III) ion cannot act as LRET-acceptor due to a missing spectral overlap. We plan to look directly at the Yb(III) luminescence and monitor alterations in a (possible) self-quenching like in the case of Pr(III) or Nd(III) in order to shed light on this aspect (the required instrumentation for time-resolved NIR luminescence detection is being set up at the moment in our lab).

The host lattice in combination with the sensitizer as well as activator properties (lattice matching, lattice phase) are not the only possible parameters to be checked in the course of minimizing the intermixing between core and shell. As well, the synthesis condition or the chemical properties of the shell(s) need to be considered, e.g., using CaF_2_ shells [17]. Another possible influence parameter could be the composition of the solvent mixture, e.g., the amount of oleic acid (“oleic acid etching”). For synthesis in octadecene the amount of oleic acid as well as the pH of the reaction solution had a distinct effect on the shape and growth of the nanoparticles. In addition, the reaction time will be of importance [55,61,62]. In the present work we have carried out the synthesis under constant conditions with respect to solvent/surfactant ratio and reaction time. Moreover, we used Therminol^®^ instead of octadecene. But in the future, these parameters may be tested. We have performed first experiments, in which we synthesized the shell(s) at lower temperatures (core synthesis at 320 °C and shell synthesis at 205 °C), but first results with respect to particle size increase or monodispersity of the nanoparticles were unsatisfactory, e.g., it seemed, that in the synthesis step of shell growth, the precursor materials formed competing seeds leading to a second generation of nanoparticles. Here, modifications in the synthesis, e.g., parameters like the addition rate of precursor materials, will be tested in future work. Additional work is in progress, in which different core and shell lattices are used (e.g., Sc(III) in the core and Y(III) or Gd(III) in the shells or Ca(II) in the outer shell). With an improved understanding of the intermixing process and how to minimize (or eliminate) it, UCNP with a higher brightness (and quantum yields) could be obtained and will make this class of optical probes even more attractive for applications in life sciences.

## Figures and Tables

**Table 1:** Overview of the sets and their sample composition with the corresponding particle sizes and their insulation shell thickness. Each set has its respective reference samples in which the LRET-acceptor is absent. The diameters are derived from the TEM images. Only the important nanoparticles for the determination of the insulation shell thickness have been examined. TEM images are shown in Figure 2 and in the Appendix A, Figure A1. Acceptor and donor doping are 20 mol% and 5 mol%, respectively, in percentage to the total amount of trivalent cations in the nanocrystal.

**Set Y300 ** **(NaYF_4_; ϑ = 320 °C)**	**Sample Composition**	**Diameter of Core-Shell (CS) Samples/nm**	**Insulation Shell Thickness/nm**
Y300 Ref CS	NaYF_4_ @ NaYF_4_:Eu	-/-	-/-
Y300 L0 CS ^1^	NaYF_4_:Pr @ NaYF_4_:Eu	7.7 ± 1.5	0
Y300 L1 CSS ^1^	NaYF_4_:Pr @ NaYF_4_ @ NaYF_4_:Eu	6.5 ± 1.3	1.4 ± 0.7
Y300 L2 CSS ^1^	vide supra	7.7 ± 1.4	2.0 ± 0.8
Y300 L3 CSS ^1^	vide supra	15.7 ± 1.0	6.0 ± 0.5
**Set Gd300** **(NaGdF_4_; ϑ = 320 °C)**	**Sample Composition**	**Diameter of Core-Shell (CS) Samples/nm**	**Insulation Shell Thickness/nm**
Gd300 Ref CS	NaGdF_4_ @ NaGdF_4_:Eu	-/-	-/-
Gd300 L0 CS ^2^	NaGdF_4_:Nd @ NaGdF_4_:Eu	8.9 ± 1.5	0
Gd300 L1 CSS ^2^	NaGdF_4_:Nd @ NaGdF_4_ @ NaGdF_4_:Eu	7.1 ± 0.4	0.7 ± 0.5
Gd300 L2 CSS ^3^	vide supra	10.8 ± 1.5	1.0 ± 1.0
Gd300 L3 CSS ^3^	vide supra	14.4 ± 1.5	2.8 ± 1.0

^1^ Common core for all samples of Y300 set with a core diameter of 3.7 ± 0.4 nm. ^2^ Common core for Gd300 L0 CS and L1 CSS with a core diameter of 5.7 ± 0.9 nm. ^3^ Common core for Gd300 L2 CSS and L3 CSS with a core diameter of 8.7 ± 1.2 nm.

**Table 2:** Set Y300 (NaYF_4_:Pr_20%_ @ NaYF_4_ @ NaYF_4_:Eu_5%_): Comparison of the insulation shell thickness, the average acceptor number, decay times *τ,* and LRET efficiencies *E*_LRET;_ evaluation of the Eu(III) luminescence at 616 nm (^5^D_0_ → ^7^F_2_) and of the Pr(III) luminescence at 608 nm (^1^D_2_ → ^3^H_4_) using Equations (1)–(4) (λ_ex_ = 465 nm) ^1^.

Set Y300	Ref CS	L3 CSS	L2 CSS	L1 CSS	L0 CS
Insulation shell thickness/nm	-/-	6.0	2.0	1.4	0
#acceptors	-/-	0.5	0.9	1.1	1.4
Eu(III) PL decay time τ/µs	4540	1950	1089	928	624
*E*_LRET_		0.57	0.76	0.80	0.86
Pr(III) PL decay time *τ_AD_*/µs (for CSS, w/Eu(III))	-/-	1.9	1.0	0.3	0.3
Pr(III) PL decay time *τ_A_*/µs (for CS, w/o Eu(III))		1.3	0.3	0.03	0.08
Pr(III) PL enhancement by Eu(III) presence		1.5	3	11	4

^1^ #acceptors within a 3D sphere with the radius of *R*_0_(Eu/Pr) = 8.2 Å. Detailed regression parameters are shown in the Appendix A, Table A2. The Pr(III) PL enhancement (= *τ_(CSS)_*/*τ*_(CS)_ = *τ_AD_*/*τ_A_*) is a factor for the increasing Pr(III) PL decay times being induced by LRET from Eu(III). (The enhancement factors of the Pr(III) PL decay times in the wavelength range of 530 nm, see Figure 3d, are given in detail in the Appendix A, Table A3).

**Table 3:** Set Gd300: Comparison of the insulation shell thickness, the acceptor numbers (#acceptors), Eu(III) decay times τ and LRET efficiencies *E*_LRET_, evaluation with Equations (1)–(4) of the Eu(III) luminescence at 616 nm (^5^D_0_ → ^7^F_2_) for the core-shell-shell nanoparticles (NaGdF_4_:Nd_20%_ @ NaGdF_4_ @ NaGdF_4_:Eu_5%_ nanoparticles), λ_ex_ = 465 nm ^1^.

Set Gd300	Ref CS	L3 CSS	L2 CSS	L1 CSS	L0 CS
Insulation shell thickness/nm	-/-	2.8	1.0	0.7	0
#acceptors	-/-	0.4	0.6	1.3	1.9
Eu(III) PL decay time *τ*/µs	2814	1505	1156	507	233
*E*_LRET_		0.47	0.59	0.82	0.92

^1^ Acceptor concentration = #acceptors within a 3D sphere with the radius of *R*_0_(Eu/Nd) = 8.53 Å. Theoretical evaluation has been performed with the FRET derived model equation. Detailed regression parameters are shown in the Appendix A, Table A4.

**Table 4:** Set Gd300: Evaluation of the Nd(III) PL decay curves with Equation (1), for PL decays in Figure 5b resulting from the major Nd(III) PL emission peaks at 795 nm and 805 nm (^2^H_9/2_ & ^4^F_5/2_ → ^4^I_9/2_), (λ_ex_ = 465 nm) ^1^.

Gd300	Core-Shell: no Eu(III)		Core-Shell-Shell: with Eu(III)		Nd(III) PL Enhancement
	*τ_A_*/µs	Heterogeneity Parameter *β*	*τ_AD_*/µs	Heterogeneity Parameter *β*	*τ*_(CSS)_/*τ*_(CS)_ = *τ_AD_*/*τ_A_*
L3	16.0 ± 0.2	0.64 ± 0.01	19.3 ± 3.7 1036 ± 166	0.51 ± 0.04 0.78 ± 0.06	1.2 65
L1	6.3 ± 0.1	0.86 ± 0.01	12.0 ± 2.5 374 ± 72	0.68 ± 0.08 0.64 ± 0.05	1.9 59

^1^ The CSS samples are listed with two decay times due to the abrupt change in the slope, compare inset in Figure 5b. Due to the significant change of the slope, the CSS samples have been analyzed twice with Equation (1). The shorter decay times (below 100 µs) correspond to the dashed regression curves in Figure 5 and refer to the points before the slope change [Nd(III) PL decay curve, without the influence of Eu(III)]. The longer decay times (larger than 100 µs) correspond to the solid regression curves in Figure 5b and refer to the points behind the slope change [Nd(III) PL decay curve with the influence of Eu(III)].

## Appendix A

**Table A1:** Overview of additional subsets and a choice of samples that were investigated by TEM: sets and their samples with the corresponding particle sizes and insulation shell thickness. Each set has its respective reference samples in which the LRET-acceptor is absent. The diameters are derived from the TEM images. Set Y300-UCNP is the same set as set Y300, except that the doping ratio on the core was changed to introduce the Yb-to-Pr upconversion pair instead of Pr(III) doping only. Set Gd200 was prepared exactly the same way as set Gd300 except for the decreased synthesis temperatures of 220 °C or 205 °C. Observation for set Y300-UCNP: The insulation layer thickness increases upon shell precursor addition. However, this trend is not as significant as expected. Observation for set Gd200: The insulation layer thickness does not increase, but it decreases. We relate that to an additional nucleation reaction during shell precursor addition. This additional nucleation reaction may be a consequence of the reduced temperature, which leads maybe to slower decomposition kinetics of the precursor material, so that the critical nucleation concentration can be exceeded. As a consequence, new nucleation seeds are generated on which the additional material starts growing instead of the provided core UCNPs that should actually serve as seeds.

**Set Y300-UCNP** **(NaYF_4_; ϑ = 320 °C)**	**Sample Composition**	**Diameter of Core-Shell (CS) ** **Samples/nm**	**Insulation Shell ** **Thickness/nm**
Y300-UCNP L0 CS ^1^	NaYF_4_:Yb_18%_, Pr_2%_ @ NaYF_4_:Eu_5%_	9.3 ± 2.2	→ 0
Y300-UCNP L1 CSS ^1^	NaYF_4_:Yb_18%_, Pr_2%_ @ NaYF_4_ @ NaYF_4_:Eu_5%_	9.5 ± 2.7	→ 0.7
Y300-UCNP L2 CSS ^2^	as above	26 ± 1.7 (87%) 15 ± 0.9 (13%)	→ 9.3 → 3.8
Y300-UCNP L3 CSS ^2^	as above	14.2 ± 4.5	→ 3.4
**Set Gd200** **(NaGdF_4_; ϑ = 220 °C)**	**Sample Composition**	**Diameter of Core-Shell (CS) Samples/nm**	**Insulation Shell Thickness/nm**
Gd200 L0 CS ^3^	NaGdF_4_:Nd @ NaGdF_4_:Eu	3.9 ± 0.3	→ 0
Gd200 L1 CSS ^3^	NaGdF_4_:Nd @ NaGdF_4_ @ NaGdF_4_:Eu	3.8 ± 0.4	→ (−) 3.4
Gd200 L2 CSS ^4^	as above	4.1 ± 0.5	→ (−) 3.3
Gd200 L3 CSS ^4^	as above	4.5 ± 0.6	→ (−) 3.1

^1^ Common core for Y300-UCNP samples L0 CS and L1 CS with a core diameter of 8.2 ± 2.5 nm. ^2^ Common core for Y300-UCNP samples L2 CS and L3 CS with a core diameter of 7.5 ± 0.7 nm. ^3^ Common core for Gd200 L0 CS and L1 CSS with a core diameter of 10.6 ± 2.0 nm. ^4^ Common core for Gd200 L2 CS and L3 CSS with a core diameter of 10.9 ± 1.7 nm.

**Table A2:** Set Y300: Detailed regression parameter for the LRET model (Equation (2)). Upper part: The Eu(III) PL decay times and the Pr(III) PL decay times (with and without Eu(III)). Bottom part: Enhancement factors. Nanoparticle composition = NaYF_4_:Pr_20%_ @ NaYF_4_ @ NaYF_4_:Eu_5%_, supplementary material to Table 2, *α* and *β* are heterogeneity parameters, λ_ex_ = 465 nm.

Eu(III) Luminescence at 616 nm (^5^D_0_ → ^7^F_2_)						
Ref CS (Donor Only)	*τ* _(Donor)_/µs	±Er	*β*	±Er		
	4540.11	37.65	0.90	0.01		
	**#acceptor**	**±Er**	***γ***	**±Er**	***α***	**±Er**
L0 CS	1.35	0.01	1.20	0.01	1.00	0.02
L1 CSS	1.07	0.02	0.95	0.02	1.00	0.04
L2 CSS	0.95	0.01	0.84	0.01	1.00	0.03
L3 CSS	0.53	0.01	0.47	0.01	1.00	0.02
	***τ*/µs**	**±Er**	***β***	**±Er**		
Ref CS	4540.11	37.65	0.90	0.01		
L3 CSS	1949.52	18.22	0.74	0.01		
L2 CSS	1089.24	11.53	0.68	0.01		
L1 CSS	928.10	10.09	0.66	0.01		
L0 CS	623.73	16.29	0.60	0.01		
**Pr(III) Luminescence at 608 nm (^1^D_2_ → ^3^H_4_)** **[CSS Samples, with Eu(III)]**						
	***τ_AD_*/µs**	**±Er**	***β_AD_***	**±Er**		
L3 CSS	1.94	0.04	0.66	0.01		
L2 CSS	1.02	0.06	0.6	0.02		
L1 CSS	0.34	0.06	0.51	0.03		
L0 CS	0.32	0.05	0.51	0.03		
**Pr(III) Luminescence at 608 nm (^1^D_2_ → ^3^H_4_)** **[CS Samples → No Eu(III)]**						
	***τ_A_*/µs**	**±Er**	***β_A_***	**±Er**		
L3 CS	1.26	0.01	0.59	1.26		
L2 CS	0.33	0.01	0.49	0.33		
L1 CS	0.03	0.01	0.39	0.03		
L0 C	0.08	0.04	0.52	0.08		
**Enhancement Factor for *τ*_(CSS/AD)_/*τ*_(CS/A)_ of Pr(III) Luminescence** **at 608 nm (^1^D_2_ → ^3^H_4_)**						
L3 CSS/CS	1.94 µs/1.26 µs → 1.5					
L2 CSS/CS	1.02 µs/0.33 µs → 3.1					
L1 CSS/CS	0.34 µs/0.03 µs → 11.3					
L0 CS/C	0.32 µs/0.08 µs → 4.0					

**Table A3:** Set Y300: Detailed regression parameter for stretched exponential model (Equation (1)) Pr(III) PL decay times *τ* of Peak 1 at 524 nm (^3^P_1_ → ^3^H_5_) and Peak 2 at 540 nm (^3^P_0_ → ^3^H_5_) with λ_ex_ = 465 nm (NaYF_4_:Pr_20%_ @ NaYF_4_ @ NaYF_4_:Eu_5%_ nanoparticles). Additional material for Figure 3 and Table 2.

Set Y300	Core-Shell: without Eu(III)		Core-Shell-Shell: with Eu(III)		Pr(III) PL Enhancement *τ*_(CSS/AD)_/*τ*_(CS/A)_
	*τ_A_*/µs	Heterogeneity Parameter *β_A_*	*τ_AD_*/µs	Heterogeneity Parameter *β_AD_*	
Peak 1: L3	1.51 ± 0.01	0.61 ± 0.01	1.91 ± 0.02	0.64 ± 0.01	1.3
Peak 1: L2	0.35 ± 0.01	0.49 ± 0.01	0.88 ± 0.01	0.56 ± 0.01	2.5
Peak 1: L1	0.02 ± 0.01	0.34 ± 0.02	0.37 ± 0.02	0.51 ± 0.01	17
Peak 1: L0	0.01 ± 0.01	0.35 ± 0.04	Not measured	Not measured	
Peak 2: L3	1.52 ± 0.01	0.62 ± 0.01	1.91 ± 0.02	0.64 ± 0.01	1.3
Peak 2: L2	0.37 ± 0.01	0.49 ± 0.01	1.01 ± 0.03	0.60 ± 0.01	2.7
Peak 2: L1	0.01 ± 0.01	0.43 ± 0.03	0.50 ± 0.03	0.57 ± 0.01	50
Peak 2: L0	0.01 ± 0.01	0.51 ± 0.06	Not measured	Not measured	

**Table A4:** Set Gd300: Detailed regression parameter for the FRET derived model equation (Equation (2)) of the Eu(III) decay times. Nanoparticle composition = NaGdF_4_:Nd_20%_ @ NaGdF_4_ @ NaGdF_4_:Eu_5%_, additional material to Table 3. *α* and *β* are heterogeneity parameters.

Eu(III) Luminescence at 616 nm (^5^D_0_ → ^7^F_2_), λ_ex_ = 465 nm						
	***τ*_(Donor)_/µs**	**±Er**	***β***	**±Er**		
Ref CS (Donor only)	2813.85	22.00	0.82	0.01		
	**#acceptor**	**±Er**	***γ***	**±Er**	***α***	**±Er**
L0 CS	1.90	0.02	1.68	0.02	1.00	0.02
L1 CSS	1.25	0.02	1.11	0.02	1.00	0.02
L2 CSS	0.57	0.01	0.51	0.01	1.00	0.02
L3 CSS	0.37	0.01	0.33	0.01	1.00	0.03
	***τ*** **/µs**	**±Er**	***β***	**±Er**		
Ref CS	2813.85	22.00	0.82	0.01		
L3 CSS	1504.81	13.52	0.73	0.01		
L2 CSS	1155.81	9.73	0.72	0.01		
L1 CSS	506.79	5.97	0.65	0.01		
L0 CS	232.70	5.732	0.59	0.01		

## References

[1] Zhu X., Zhang J., Liu J., Zhang Y. (2019). Recent Progress of Rare-Earth Doped Upconversion Nanoparticles: Synthesis, Optimization, and Applications. Adv. Sci..

[2] Tessitore G., Mandl G.A., Brik M.G., Park W., Capobianco J.A. (2019). Recent insights into upconverting nanoparticles: Spectroscopy, modeling, and routes to improved luminescence. Nanoscale.

[3] Naccache R., Yu Q., Capobianco J.A. (2015). The Fluoride host: Nucleation, growth, and upconversion of lanthanide-doped nanoparticles. Adv. Opt. Mater..

[4] Würth C., Kaiser M., Wilhelm S., Grauel B., Hirsch T., Resch-Genger U. (2017). Excitation power dependent population pathways and absolute quantum yields of upconversion nanoparticles in different solvents. Nanoscale.

[5] DaCosta M.V., Doughan S., Han Y., Krull U.J. (2014). Lanthanide upconversion nanoparticles and applications in bioassays and bioimaging: A review. Anal. Chim. Acta.

[6] Chen G., Qiu H., Prasad P.N., Chen X. (2014). Upconversion nanoparticles: Design, nanochemistry, and applications in theranostics. Chem. Rev..

[7] Muhr V., Wilhelm S., Hirsch T., Wolfbeis O.S. (2014). Upconversion nanoparticles: From hydrophobic to hydrophilic surfaces. Acc. Chem. Res..

[8] Andresen E., Resch-Genger U., Schäferling M. (2019). Surface Modifications for Photon-Upconversion-Based Energy-Transfer Nanoprobes. Langmuir.

[9] Gorris H.H., Wolfbeis O.S. (2013). Photon-upconverting nanoparticles for optical encoding and multiplexing of cells, biomolecules, and microspheres. Angew. Chem.-Int. Ed..

[10] Hudry D., Busko D., Popescu R., Gerthsen D., Abeykoon A.M.M., Kübel C., Bergfeldt T., Richards B.S. (2017). Direct Evidence of Significant Cation Intermixing in Upconverting Core@Shell Nanocrystals: Toward a New Crystallochemical Model. Chem. Mater..

[11] Hudry D., Popescu R., Diaz-Lopez M., Abeykoon A.M.M., Bordet P., Gerthsen D., Howard I.A., Richards B.S. (2019). Interface disorder in large single- and multi-shell upconverting nanocrystals. J. Mater. Chem. C.

[12] Hudry D., Busko D., Popescu R., Gerthsen D., Howard I.A., Richards B.S. (2019). An enhanced energy migration strategy in upconverting nanocrystals: Color-tuning with high quantum yield. J. Mater. Chem. C.

[13] Hudry D., Howard I.A., Popescu R., Gerthsen D., Richards B.S. (2019). Structure-Property Relationships in Lanthanide-Doped Upconverting Nanocrystals: Recent Advances in Understanding Core–Shell Structures. Adv. Mater..

[14] Diogenis I.M.S., Rodrigues E.M., Mazali I.O., Sigoli F.A. (2021). Spectroscopic evidence of preferential excitation of interfacial Eu^III^ by interfacial energy transfer process on core@shell nanoparticles. J. Lumin..

[15] Liu L., Li X., Fan Y., Wang C., El-Toni A.M., Alhoshan M.S., Zhao D., Zhang F. (2019). Elemental Migration in Core/Shell Structured Lanthanide Doped Nanoparticles. Chem. Mater..

[16] Chen B., Peng D., Chen X., Qiao X., Fan X., Wang F. (2015). Establishing the structural integrity of core-shell nanoparticles against elemental migration using luminescent lanthanide probes. Angew. Chem.-Int. Ed..

[17] Dong H., Sun L.D., Li L.D., Si R., Liu R., Yan C.H. (2017). Selective cation exchange enabled growth of lanthanide core/shell nanoparticles with dissimilar structure. J. Am. Chem. Soc..

[18] Goldschmidt V.M. (1926). Die Gesetze der Krystallochemie. Naturwissenschaften.

[19] Bastian P.U., Nacak S., Roddatis V., Kumke M.U. (2020). Tracking the motion of lanthanide ions within core–shell–shell NaYF_4_ nanocrystals via resonance energy transfer. J. Phys. Chem. C.

[20] Horrocks W.D.W., Sudnick D.R. (1981). Lanthanide ion luminescence probes of the structure of biological macromolecules. Acc. Chem. Res..

[21] Wang F., Han Y., Lim C.S., Lu Y., Wang J., Xu J., Chen H., Zhang C., Hong M., Liu X. (2010). Simultaneous phase and size control of upconversion nanocrystals through lanthanide doping. Nature.

[22] Liu Q., Sun Y., Yang T., Feng W., Li C., Li F. (2011). Sub-10 nm hexagonal lanthanide-doped NaLuF 4 upconversion nanocrystals for sensitive bioimaging in vivo. J. Am. Chem. Soc..

[23] Jia H., Zhou Y., Li X., Li Y., Zhang W., Fu H., Zhao J., Pan L., Liu X., Qiu J. (2018). Synthesis and phase transformation of NaGdF_4_:Yb-Er thin films using electro-deposition method at moderate temperatures. CrystEngComm.

[24] Park Y.I., Kim H.M., Kim J.H., Moon K.C., Yoo B., Lee K.T., Lee N., Choi Y., Park W., Ling D. (2012). Theranostic probe based on lanthanide-doped nanoparticles for simultaneous in vivo dual-modal imaging and photodynamic therapy. Adv. Mater..

[25] Annapurna K., Dwivedi R.N., Buddhudu S. (2000). Energy transfer luminescence in (Eu^3+^,Nd^3+^): Tellurite glass. Opt. Mater..

[26] Sharp E.J., Weber M.J., Cleek G. (1970). Energy transfer and fluorescence quenching in Eu- and Nd-doped silicate glasses. J. Appl. Phys..

[27] Abad Galán L., Sobolev A.N., Skelton B.W., Zysman-Colman E., Ogden M.I., Massi M. (2018). Energy transfer between Eu^3+^ and Nd^3+^ in near-infrared emitting β-triketonate coordination polymers. Dalt. Trans..

[28] Joshi J.C., Pandey N.C., Joshi B.C., Belwal R., Joshi J. (1978). Quantum efficiency of Diffusion-Limited Energy Transfer from Eu^3+^ → Nd^3+^ in Borate Glass. J. Solid State Chem..

[29] Kandpal H.C., Tripathi H.B. (1979). Non-radiative energy transfer from Eu^3+^ to Pr^3+^ and Eu^3+^ to Er^3+^ in DMSO. Solid State Commun..

[30] Chen Y., Wang J., Liu C., Tang J., Kuang X., Wu M., Su Q. (2013). UV-Vis-NIR luminescence properties and energy transfer mechanism of LiSrPO_4_:Eu^2+^, Pr^3+^ suitable for solar spectral convertor. Opt. Express.

[31] Li Y., Wang J., Wang X.M., Pan F., Zhou T., Xie R.J. (2017). Colour tuning via crystalline site-selected energy transfer in a Sr_2_SiO_4_:Eu^2+^,Pr^3+^ phosphor. J. Mater. Chem. C.

[32] Da Gama A.A.S., De Sá G.F., Porcher P., Caro P. (1981). Energy levels of Nd^3+^ in LiYF_4_. J. Chem. Phys..

[33] Runowski M., Woźny P., Martín I.R., Lavín V., Lis S. (2019). Praseodymium doped YF_3_:Pr^3+^ nanoparticles as optical thermometer based on luminescence intensity ratio (LIR)—Studies in visible and NIR range. J. Lumin..

[34] Hao S., Shao W., Qiu H., Shang Y., Fan R., Guo X., Zhao L., Chen G., Yang C. (2014). Tuning the size and upconversion emission of NaYF_4_:Yb^3+^/Pr^3+^ nanoparticles through Yb^3+^ doping. RSC Adv..

[35] Dieke G.H., Crosswhite H.M. (1963). The Spectra of the Doubly and Triply Ionized Rare Earths. Appl. Opt..

[36] Carnall W.T., Fields P.R., Rajnak K. (1968). Electronic Energy Levels in the Trivalent Lanthanide Aquo Ions. I. Pr^3+^, Nd^3+^, Pm^3+^, Sm^3+^, Dy^3+^, Ho^3+^, Er^3+^, and Tm^3+^. J. Chem. Phys..

[37] Carnall W.T., Fields P.R., Rajnak K. (1968). Electronic Energy Levels of the Trivalent Lanthanide Aquo Ions. IV. Eu^3+^. J. Chem. Phys..

[38] Nakazawa E., Shionoya S. (1967). Energy transfer between trivalent rare-earth ions in inorganic solids. J. Chem. Phys..

[39] Hesse J., Klier D.T., Sgarzi M., Nsubuga A., Bauer C., Grenzer J., Hübner R., Wislicenus M., Joshi T., Kumke M.U. (2018). Rapid Synthesis of Sub-10 nm Hexagonal NaYF_4_-Based Upconverting Nanoparticles using Therminol® 66. ChemistryOpen.

[40] Aebischer A., Hostettler M., Hauser J., Krämer K., Weber T., Güdel H.U., Bürgi H.B. (2006). Structural and spectroscopic characterization of active sites in a family of light-emitting sodium lanthanide tetrafluorides. Angew. Chem.-Int. Ed..

[41] Szefczyk B., Roszak R., Roszak S. (2014). Structure of the hexagonal NaYF_4_ phase from first-principles molecular dynamics. RSC Adv..

[42] Grzechnik A., Bouvier P., Mezouar M., Mathews M.D., Tyagi A.K., Köhler J. (2002). Hexagonal Na1.5Y1.5F6 at high pressures. J. Solid State Chem..

[43] Krämer K.W., Biner D., Frei G., Güdel H.U., Hehlen M.P., Lüthi S.R. (2004). Hexagonal Sodium Yttrium Fluoride Based Green and Blue Emitting Upconversion Phosphors. Chem. Mater..

[44] Burns J.H. (1965). Crystal Structure of Hexagonal Sodium Neodymium Fluoride and Related Compounds. Inorg. Chem..

[45] Mackenzie L.E., Goode J.A., Vakurov A., Nampi P.P., Saha S., Jose G., Millner P.A. (2018). The theoretical molecular weight of NaYF_4_: RE upconversion nanoparticles. Sci. Rep..

[46] Wu X., Zhan S., Han J., Liu Y. (2021). Nanoscale Ultrasensitive Temperature Sensing Based on Upconversion Nanoparticles with Lattice Self-Adaptation. Nano Lett..

[47] Valeur B. (2002). Molecular Fluorescence—Principles and Applications.

[48] Benny Lee K.C., Siegel J., Webb S.E.D., Lévêque-Fort S., Cole M.J., Jones R., Dowling K., Lever M.J., French P.M.W. (2001). Application of the stretched exponential function to fluorescence lifetime imaging. Biophys. J..

[49] Li K.Y., Liu L.Y., Wang R.Z., Xiao S.G., Zhou H., Yan H. (2014). Broadband sensitization of downconversion phosphor YPO_4_ by optimizing TiO_2_ substitution in host lattice co-doped with Pr^3+^-Yb^3+^ ion-couple. J. Appl. Phys..

[50] Chen X.P., Huang X.Y., Zhang Q.Y. (2009). Concentration-dependent near-infrared quantum cutting in NaYF_4_:Pr^3+^, Yb^3+^ phosphor. J. Appl. Phys..

[51] Binnemans K. (2015). Interpretation of europium(III) spectra. Coord. Chem. Rev..

[52] Rabouw F.T., Prins P.T., Norris D.J. (2016). Europium-Doped NaYF_4_ Nanocrystals as Probes for the Electric and Magnetic Local Density of Optical States throughout the Visible Spectral Range. Nano Lett..

[53] Bro R. (1997). PARAFAC tutorial and applications. Chemom. Intell. Lab. Syst..

[54] Görller-Walrand C., Binnemans K., Gschneidner K.A., Eyring L. (1996). Rationalization of Crystal-Field Parametrization. Handbook on the Physics and Chemistry of Rare Earths.

[55] Voss B., Haase M. (2013). Intrinsic focusing of the particle size distribution in colloids containing nanocrystals of two different crystal phases. ACS Nano.

[56] Rinkel T., Nordmann J., Raj A.N., Haase M., Val’kovskii M.D., Sobolev B.P., Park J., Hwang N., Hyeon T., Cohen B.E. (2014). Ostwald-ripening and particle size focussing of sub-10 nm NaYF_4_ upconversion nanocrystals. Nanoscale.

[57] Dühnen S., Haase M. (2015). Study on the Intermixing of Core and Shell in NaEuF_4_/NaGdF_4_ Core/Shell Nanocrystals. Chem. Mater..

[58] Rinkel T., Raj A.N., Dühnen S., Haase M. (2016). Synthesis of 10 nm β-NaYF_4_:Yb, Er/NaYF_4_ core/shell upconversion nanocrystals with 5 nm particle cores. Angew. Chem.-Int. Ed..

[59] Huheey J.E., Keiter E.A., Keiter R.L., Steudel R. (2012). Anorganische Chemie—Prinzipien von Struktur und Reaktivität.

[60] Pfiffner O.A., Engi M., Schlunegger F., Mezger K., Diamond L. (2016). Erdwissenschaften. Erdwissenschaften.

[61] Oh N., Shim M. (2016). Metal oleate induced etching and growth of semiconductor nanocrystals, nanorods, and their heterostructures. J. Am. Chem. Soc..

[62] Liu D., Xu X., Du Y., Qin X., Zhang Y., Ma C., Wen S., Ren W., Goldys E.M., Piper J.A. (2016). Three-dimensional controlled growth of monodisperse sub-50 nm heterogeneous nanocrystals. Nat. Commun..

